# Diffraction data from aerosolized Coliphage PR772 virus particles imaged with the Linac Coherent Light Source

**DOI:** 10.1038/s41597-020-00745-2

**Published:** 2020-11-19

**Authors:** Haoyuan Li, Reza Nazari, Brian Abbey, Roberto Alvarez, Andrew Aquila, Kartik Ayyer, Anton Barty, Peter Berntsen, Johan Bielecki, Alberto Pietrini, Maximilian Bucher, Gabriella Carini, Henry N. Chapman, Alice Contreras, Benedikt J. Daurer, Hasan DeMirci, Leonie Flűckiger, Matthias Frank, Janos Hajdu, Max F. Hantke, Brenda G. Hogue, Ahmad Hosseinizadeh, Mark S. Hunter, H. Olof Jönsson, Richard A. Kirian, Ruslan P. Kurta, Duane Loh, Filipe R. N. C. Maia, Adrian P. Mancuso, Andrew J. Morgan, Matthew McFadden, Kerstin Muehlig, Anna Munke, Hemanth Kumar Narayana Reddy, Carl Nettelblad, Abbas Ourmazd, Max Rose, Peter Schwander, M. Marvin Seibert, Jonas A. Sellberg, Raymond G. Sierra, Zhibin Sun, Martin Svenda, Ivan A. Vartanyants, Peter Walter, Daniel Westphal, Garth Williams, P. Lourdu Xavier, Chun Hong Yoon, Sahba Zaare

**Affiliations:** 1 SLAC National Accelerator Laboratory, 2575 Sand Hill Road, Menlo Park, California 94025 USA; 2 Physics Department, Stanford University, 450 Serra Mall, Stanford, California 94305 USA; 3 Arizona State University, 1001S. McAllister Avenue, Tempe, AZ 85287 USA; 4 ARC Centre of Excellence in Advanced Molecular Imaging, La Trobe University, Bundoora, VIC 3086 Australia; 5 Max Planck Institute for the Structure and Dynamics of Matter, Luruper Chaussee 149, 22761 Hamburg, Germany; 6 Center for Free Electron Laser Science, DESY, Notkestrasse 85, 22607 Hamburg, Germany; 7 DESY, Photon Science, Notkestrasse 85, 22607 Hamburg, Germany; 8 European XFEL, Holzkoppel 4, 22869 Schenefeld, Germany; 9 Laboratory of Molecular Biophysics, Department of Cell and Molecular Biology, Uppsala University, Husargatan 3 (Box 596), SE-751 24 Uppsala, Sweden; 10 Brookhaven National Laboratory, Bldg 535B, Upton, NY 11973 USA; 11 Centre for Ultrafast Imaging, Luruper Chaussee 149, 22761 Hamburg, Germany; 12 Diamond Light Source, Harwell Science & Innovation Campus, Didcot, OX11 0DE United Kingdom; 13 Stanford PULSE Institute, 2575 Sand Hill Road, Menlo Park, California 94025 USA; 14 Koc University, Rumelifeneri, Sariyer Rumeli Feneri Yolu, 34450 Sariyer/Istanbul, Turkey; 15 Lawrence Livermore National Laboratory, 7000 East Avenue, L-452, Livermore, California 94550 USA; 16 The European Extreme Light Infrastructure, Institute of Physics, Academy of Sciences of the Czech Republic, Za Radnicic 835, 25241 Dolní Břežany, Czech Republic; 17 University of Wisconsin Milwaukee, 3135N. Maryland Ave, Milwaukee, Wisconsin 53211 USA; 18 Department of Applied Physics, KTH Royal Institute of Technology, AlbaNova University Center, KTH Royal Institute of Technology, S-106 91 Stockholm, Sweden; 19 Department of Physics, National University of Singapore, 14 Science Drive 4, Blk S1A, Level 2, S1A-02-07, Lee Wee Kheng Building, Singapore, 117557 Singapore; 20 Department of Chemistry and Physics, La Trobe Institute for Molecular Science, La Trobe University, Melbourne, Victoria 3086 Australia; 21 ARC Centre of Excellence in Advanced Molecular Imaging, School of Physics, University of Melbourne, Parkville, Victoria 3010 Australia; 22 Photon Science Division, Paul Scherrer Institute, CH-5232 Villigen PSI, Switzerland; 23 NRNU MEPhI, Kashirskoe shosse 31, 115409 Moscow, Russia

**Keywords:** Structural biology, Biological physics, Imaging and sensing

## Abstract

Single Particle Imaging (SPI) with intense coherent X-ray pulses from X-ray free-electron lasers (XFELs) has the potential to produce molecular structures without the need for crystallization or freezing. Here we present a dataset of 285,944 diffraction patterns from aerosolized Coliphage PR772 virus particles injected into the femtosecond X-ray pulses of the Linac Coherent Light Source (LCLS). Additional exposures with background information are also deposited. The diffraction data were collected at the Atomic, Molecular and Optical Science Instrument (AMO) of the LCLS in 4 experimental beam times during a period of four years. The photon energy was either 1.2 or 1.7 keV and the pulse energy was between 2 and 4 mJ in a focal spot of about 1.3 *μ*m x 1.7 *μ*m full width at half maximum (FWHM). The X-ray laser pulses captured the particles in random orientations. The data offer insight into aerosolised virus particles in the gas phase, contain information relevant to improving experimental parameters, and provide a basis for developing algorithms for image analysis and reconstruction.

## Abstract (summary)

**Table:** 

Measurement(s)	X-ray diffraction data
Technology Type(s)	free electron laser
Sample Characteristic - Organism	Enterobacteria phage PR772
Sample Characteristic - Location	contiguous United States of America

Machine-accessible metadata file describing the reported data: 10.6084/m9.figshare.13070546

## Background & Summary

Since the establishment of the single particle initiative^1^, several experiments have been conducted at the Linac Coherent Light Source (LCLS) to identify and resolve experimental challenges in high-resolution Single Particle Imaging (SPI) experiments^2,3^. Coliphage PR772 viruses were utilized extensively in these experiments as the standard control sample due to its high structural homogeneity, uniformity, stability, suitable particle concentration in solution, and the ability to be aerosolized for injection into the LCLS beam using aerosol injector technology^2,4,5^.

An initial dataset from experiments using Coliphage PR772 performed at the LCLS in 2015 was published in 2017^6^ to assist in the development of analysis methods. Since that experiment, several additional experiments have been performed to push the method to higher resolutions and carry out testing of different aerosolization and sample delivery methods. Coliphage PR772 was also used as a standard reference sample in those subsequent experiments. This provides an opportunity to investigate the influence of experiment conditions on data quality and to check the reproducibility of SPI experiments in addition to obtaining higher resolution data. The purpose of this paper is to describe data from these additional experiments^7^.

Four experiment runs with PR772 have been performed in the years from 2015 to 2018 (amo87215, amo06516, amo11416, amox34117). This paper summarizes the data collected in those experiments, the experimental conditions, and classification results for single-hit diffraction patterns. We provide appropriate metadata for interpreting the images including: photon energy, X-ray pulse energy and length, position of each pixel relative to the interaction region, bad-pixel mask, the run number and index for all classified hits and the run number and index for all single hits. Analysis of diffraction patterns from real experiments with a variety of experimental configurations can potentially facilitate the development of a robust data processing pipeline for the processing of experimental single particle diffraction data.

## Methods

In single particle diffraction-before-destruction imaging experiments^8^ a sample, usually biological, is introduced into the focus of an XFEL beam where the X-ray fluence is high enough to destroy the sample with each pulse, however the pulse duration is so short that this does not happen before a 2D diffraction pattern is formed. For samples that are small and non-crystaline, such as individual viruses or biomolecules, the scattered signal containing structural information is weak and often in a photon counting regime. However, using a continuously replenished stream of identical particles in random orientations, a 3D diffraction volume with sufficient signal-to-noise for structure determination can be composed from the individual measurements provided the particle orientations can be determined and sufficient diffraction patterns have been measured. The 3D diffraction volume has a higher resolution than any given single diffraction pattern and can be inverted to form a real space representation of the average particle. Details of the methods used for sample preparation, sample delivery, instrumentation, and preliminary data analysis are described below.

### Sample preparation

**Fig. 1:**
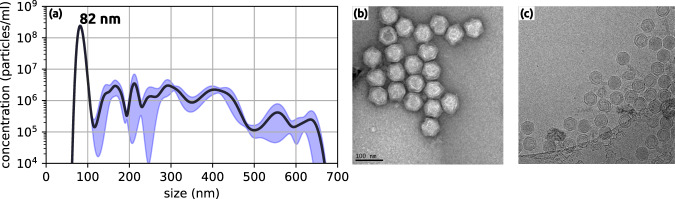
Sample verification of PR772 used in AMOX34117. (**a**) Nanoparticle tracking analysis conducted on PR772 to determine concentration and size. The first and dominant peak is at 82 nm, with a concentration of (2.4 ± 0.09) × 10^8^ particles/ml. The standard error is shown in blue. Note: the sample was diluted by 10^4^ to allow for a more accurate peak determination. (**b**) Negative stained transmission electron microscopy image of PR772. (**c**) Cryogenic transmission electron microscopy imaging of PR772 using a Krios electron microscope.

PR772 bacteriophage growth and purification was performed as previously described^6^. For completeness, we provide a brief overview of the process here. The samples were grown overnight in E. coli, then cultured onto hard agar plates and incubated overnight at 37 °C. The samples were then scraped from the plates, placed in a storage buffer consisting of 50 mM Tris, 100 mM NaCl, 1 mM MgSO4, 1 mM EDTA at a pH of 8.0, and incubated on a rocker overnight at 4 °C. The mixture was centrifuged at 8,000 g for 30 min to remove the agar and cell debris. The supernatant was then collected and filtered through a 0.2 *μ*m filter. Viral particles were separated from the solution by PEG precipitation with PEG 8000 (9% w/v PEG + 5.8% w/v NaCl) and left to mix overnight on a rocker at 4 °C. After mixing, the precipitate was centrifuged for 90 min at 8,000 g at 4 °C to pellet the virus. The viral pellet was then suspended in the storage buffer. A Capto-Q anion exchange column using FPLC was then applied. The sample was eluted by NaCl (typical concentrations 750 to 900 mM). Just prior to sample injection, the PR772 virus particles were transferred from the storage buffer into a volatile ammonium acetate buffer (250 mM, pH 7.5) using PD10 desalting columns (GE Healthcare). Verification of the sample was conducted using electron microscopy and nanoparticle tracking analysis as shown in Fig. 1.

### Sample delivery

**Fig. 2:**
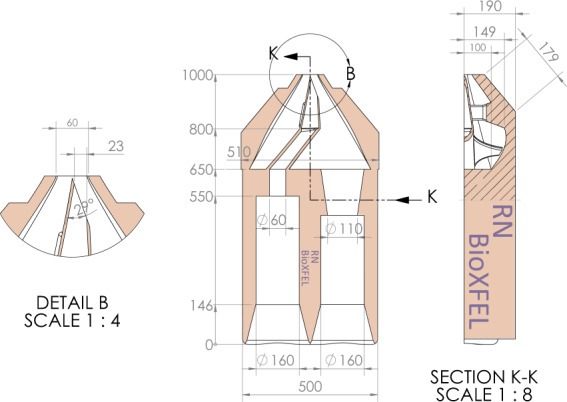
Schematic diagram of the 3D printed GDVN design used in the amox34117 measurements.

For all datasets described here, PR772 bacteriophage was aerosolized using gas dynamic virtual nozzles (GDVN)^9,10^ with helium as the nebuliser gas. For amo87215, amo06516, and amo11416 a glass GDVN nozzle was used (ground and polished with an outer diameter of 1.0 mm and an initial inner diameter of 0.78 mm). The Glass GDVN Nozzles were melted to create a much smaller inner diameter of order 15 to 20 *μ*m. For amox34117 the nozzle was 3D printed via 2-photon polymerization photo-lithography with a Nanoscribe Professional GT printer^11^. These 3D printed nozzles (shown in Fig. 2) had an asymmetric “syringe tip” design featuring an elliptical liquid orifice with minor/major axis diameters of 23 *μ*m and 68 *μ*m, respectively, and an exit gas aperture of 60 *μ*m. The virus particles were then passed through a differentially pumped skimmer that was used for pressure reduction (from atmospheric to typically 60 to 300 Pa at the exit of the skimmers). The skimmer is needed for the proper use of the particle focusing system and to limit the maximum sample chamber pressure to 4 × 10^−3^ Pa. The chamber pressure limit is required to reduce the background scattering from the carrier gas and to protect the detector from thermal drift and high voltage arcing. The samples were then focused into the sample chamberâ€™s interaction point of the X-ray instrument using an aerodynamic lens stack injector^4,5^.

### Instrumentation

**Fig. 3:**
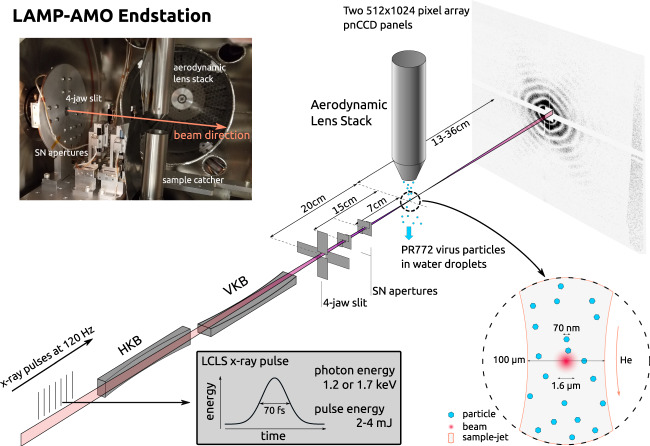
Diagram of the experimental setup including X-ray pulses, X-ray focusing (horizontal and vertical) KB mirror pair, Silicon Nitrite apertures and slits, sample delivery aerodynamic lens stack and PNCCD detector. The insert at the top left shows the inside of the sample chamber containing the apertures and slit system as well as the sample delivery system.

All four experiments were conducted at the LAMP endstation of the AMO instrument at the LCLS^12–14^. A schematic of these experiments is shown in Fig. 3. The instrument uses a pair of Boron Carbide coated Kirkpatrick-Baez (KB) mirrors capable of focusing the FEL beam to a nominal 1.5 *μ*m diameter focal spot. Wavefront sensor measurements taken in 2017 show the focused X-ray beam to be nearly Gaussian in shape with a FWHM of 1.3 *μ*m × 1.7 *μ*m (vertical × horizontal). Shot by shot X-ray pulse energies were measured with gas monitors^15^ located upstream of the AMO optics. Measured pulse energies varied between 2 and 4 mJ per pulse and are included in the metadata for each diffraction image. It is noted that the X-ray optical transport system of the AMO instrument is not perfect and has been measured to be ~40% efficient. Background scatter, from the upstream optics and residual gas in the chamber, was reduced using a beveled silicon nitride 4-jaw slit followed by two motorized 1 mm × 1 mm opening silicon nitride apertures used to reduce scatter from the 4-jaw slit. The 4-jaw slit was located ~20 cm upstream of the focus and the two apertures were located ~15 cm and ~7 cm respectively upstream of the focus. Additionally, adjustable rolled B_4_C slits were used 2.0 m upstream of the KB mirrors to define the entrance aperture of the focusing system (not shown in Fig. 3).

Initial alignment of the aerodynamic lens injector to the focal spot position was performed using the beamline alignment laser and a retractable alignment pin coated in a powdered phosphor to directly align the center of the injector with the X-ray focus. The injector was positioned 3 mm above the X-ray focus. Lateral scans of the injector were conducted for each experiment to optimize hit rates. The focus of the particle stream was found to be approximately 100 *μ*m (full width at half maximum) with variation in focal spot size depending on inlet and chamber pressures.

The samples exiting the aerodynamic lens injector and entering the X-ray interaction region of the instrument are in random orientations and also enter the interaction point at random time intervals, as the aerodynamic lens does not align the particles in any particular orientation. As the sample delivery focus was far greater than that of the X-ray pulses in width (as illustrated in the inset of Fig. 3) the majority of X-ray pulses miss the sample and do not interact with any particles. The LCLS provides 120 equally spaced X-ray pulses per second and typically _~_1% of these will intersect with a sample, depending on the sample concentration, GDVN and skimmer operating conditions.

**Fig. 4:**
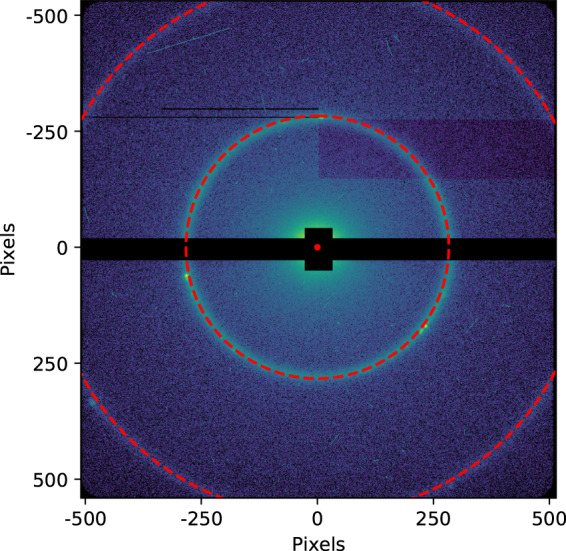
Calibration of pnCCD detector position for experiment amo11416. The detector gap, beam center, and camera length are found using the lowest diffraction rings of Silver Behenate. The central ring corresponds to a resolution of 5.84 nm ($| \overrightarrow{Q}| =0.1076\;{{\rm{\AA }}}^{-1}$), while the outer ring corresponds to a resolution of 2.92 nm ($| \overrightarrow{Q}| =0.2152\;{{\rm{\AA }}}^{-1}$).

Diffracted X-rays are collected, downstream of the interaction point on two 512 × 1024 pixel pnCCD panels^16,17^. The detector consists of two panels which are movable jointly along the X-ray beam axis, Z, and the two panels can also be moved independently vertically, Y, with respect to the horizontal gap between the two detector panels. When no particle is present in the X-ray focus the measured intensity corresponds to instrument background due to scatter from residual gas, slits, and so on; however, when a sample particle interacts with the XFEL beam a coherent diffraction pattern is additionally measured on the detector. The position of both panels and the camera length of the detector from the interaction region was determined using the known diffraction of Silver Behenate prior to each experiment. An example of such a calibration is shown in Fig. 4.

An X-ray photon energy of 1.7 keV (0.73 nm) was used for most of the experiments reported here, except for during runs 38–58 of the amo87215 experiment where an X-ray photon energy of 1.2 keV (1.03 nm) was used (other runs in amo87215 were at 1.7 keV).

Both the detector distance and the detector gap size have been optimized for the measurement of high resolution data throughout the experiments. The detector distance and the detector edge resolution for each experiment can be found in Table 1. Notice that, in amo11416, for runs 55 and 56, the gap size is different from the previous runs to reach a higher edge resolution of 2.8 nm.

**Table 1:** Summary of experiment conditions and dataset statistics.

Exp Name	AMO87215		AMO06516	AMO11416		AMOX34117
Run Range	49–58	59–78	90–143	38–50	55,56	130–236
Photon Energy (eV)	1210.6	1536.0	1656.4	1653.1		1701.6
Detector Distance (cm)	360		283	219		130
Edge Resolution (nm)	9.7	7.6	5.5	4.2	2.8	1.8
Single Hit Number	24	2450	9033	211	2450	1393
Total Hit Number	216	11230	84596	4546	11230	197667
Single/Total Ratio	11.1%	21.8%	10.7%	4.6%	21.8%	0.7%
Approx. Run Time (hr)	1.25	6.45	10.34	4.05	0.87	22.83

### Data processing

The pnCCD detector is an integrating detector that reads out the deposited charge incident on each pixel in analog-to-digital units (ADUs). Photon counting detectors cannot be used for this type of experiment due to the arrival of multiple photons in an individual pixel within the space of a few femtoseconds^18^. However, integrating detectors (such as the pnCCD) can still achieve single photon sensitivity under certain conditions. A series of corrections and calibrations are required in order to convert the data from ADUs to photon counts per pixel. In this report, we use *psana*, an LCLS software framework^19,20^, to retrieve the data, obtain the detector pixel positions, mask for bad pixels and apply various corrections to convert the ADUs into photon counts.

**Fig. 5:**
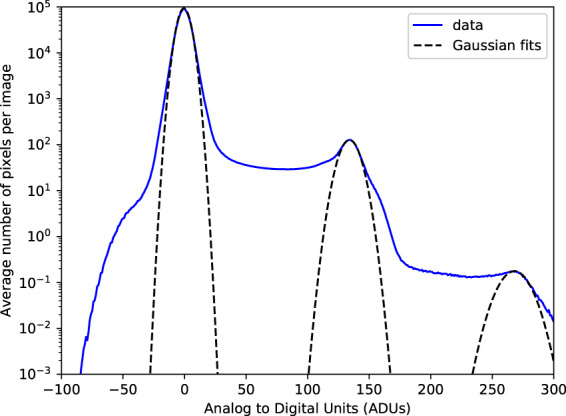
Calibration of pnCCD detector for ADUs per photon using silicon fluorescence (*Kα* = 1.74 keV) during the amo06516 experiment. Shown is a histogram of the average number of ADUs and the average number of pixels per image giving the ADU value averaged over 10,000 data frames/readouts. The fluence in the calibration was kept low so there was less than one 2 photon event per collected frame. The 1 photon peak was found to be 134 ADUs with a width of *σ* = 9.7 ADUs, while the 2 photon peak was found to be 268 ADUs with a width of *σ* = 15 ADUs. It is noted that there is significant number of pixels with ADU values between 0 and 1 photon. These events are due to charge sharing between pixels. This happens when a photon strikes close enough to the edge of a pixel that the resulting electron cloud of charge created is shared between pixels.

Corrections applied to the pnCCD data include (in order) pedestal subtraction, common-mode correction and gain correction followed by conversion to photon counts. As each photon strikes a given pixel, an electron cloud is generated in the substrate of the detector panel, with the number of electrons being proportional to the number of incident photons, the photon energy and the degree of charge sharing between neighbouring pixels. This current is then integrated to form the ADU count for that pixel. In Fig. 5 we show a histogram of the measured ADU counts from silicon fluorescence (*Kα* = 1.74 keV) after pedestal and common-mode correction (i.e. subtraction of average CCD dark current and voltage offsets). The modal ADU values corresponding to zero, one and two incident photons are situated at the peaks of the three Gaussian profiles (black dashed lines) with values of 0, 134 and 268 ADUs for a gain setting of 4, respectively. The spread in the ADU values about these peaks are due to the stochastic nature of the pedestal, gain and charge sharing processes. Thus, simple division of ADUs by the mean ADUs-per-photon yields poor photon conversion. We used a psana built-in function^19,20^ (*detector.photons*) to convert the ADUs into photon counts for each pixel which accounts for charge sharing and incident photon energy.

**Fig. 6:**
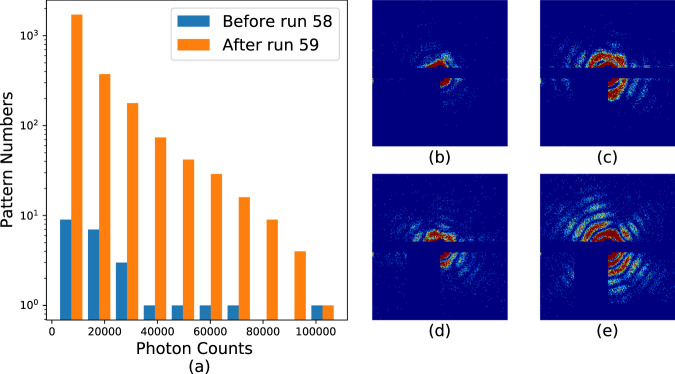
Histograms and typical single hits for experiment AMO87215. (**a**) The histogram of the total photon counts of the single hit patterns in this experiment. (**b**,**c**) are randomly selected patterns from the 1st and 3rd column in the histogram for run number less than or equal to 58. (**d**,**e**) are randomly selected patterns from the 2nd and 6th column in the histogram for run number larger than or equal to 59. The boundary is colored with the same color as that of the corresponding column. Single hit patterns are rendered with matplotlib.pyplot.imshow funcitons with color map “jet” and *vmax* = 4. Before rendering, the photon count patterns are first down-sampled 4-by-4 times.

**Fig. 7:**
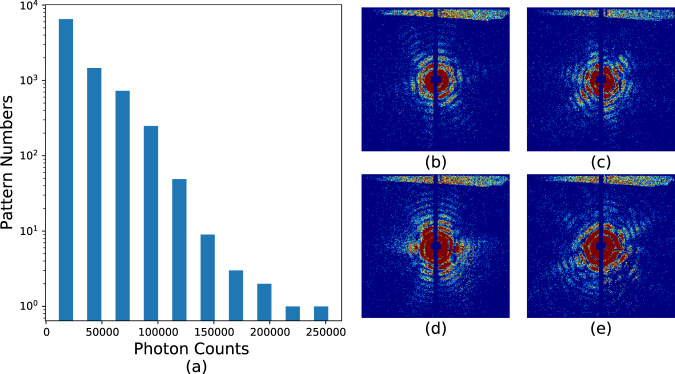
Histograms and typical single hits for experiment AMO06516.(**a**) The histogram of the total photon counts of the single hit patterns in this experiment. (**b**–**e**) Each is a random pattern selected from the 1st, 3rd, 5th and 7th column in the histogram. The boundary is colored with the same color as that of the corresponding column. Single hit patterns are rendered with matplotlib.pyplot.imshow funcitons with color map “jet” and *vmax* = *4*. Before rendering, the photon count patterns are first down-sampled 4-by-4 times.

**Fig. 8:**
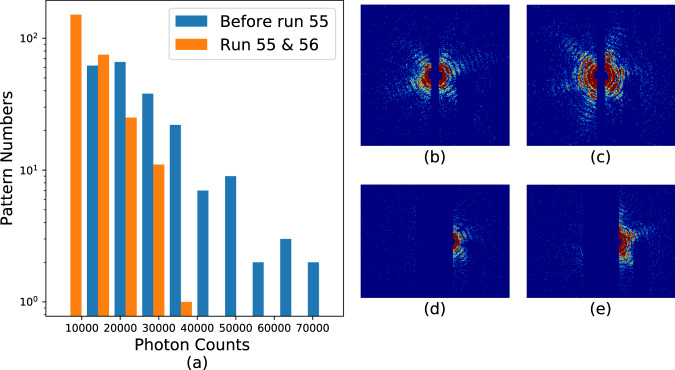
Histograms and typical single hits for experiment AMO11416. (**a**) The histogram of the total photon counts of the single hit patterns in this experiment. (**b**,**c**) are randomly selected patterns from the 2st and 6rd column in the histogram for run number less than or equal to 54. (**d**,**e**) are randomly selected patterns from the 1nd and 3th column in the histogram for run number larger than or equal to 55. The boundary is colored with the same color as that of the corresponding column. Single hit patterns are rendered with matplotlib.pyplot.imshow funcitons with color map “jet” and *vmax* = *4*. Before rendering, the photon count patterns are first down-sampled 4-by-4 times.

**Fig. 9:**
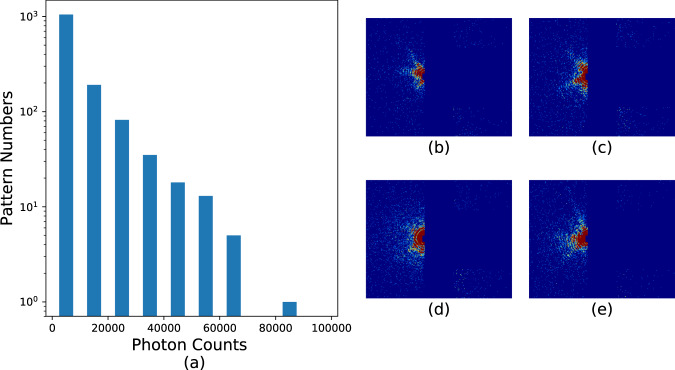
Histograms and typical single hits for experiment AMOX34117. (**a**) The histogram of the total photon counts of the single hit patterns in this experiment. (**b**–**e**) Each is a random pattern selected from the 1st, 3rd, 5th and 7th column in the histogram. The boundary is colored with the same color as that of the corresponding column. Single hit patterns are rendered with matplotlib.pyplot.imshow funcitons with color map “jet” and *vmax* = *4*. Before rendering, the photon count patterns are first down-sampled 4-by-4 times.

Hit rates in these experiments were typically _~_1% as previously mentioned. Hits are defined as frames containing discernible diffraction from the sample, which are identified as frames with significantly elevated diffraction intensity. This process is accomplished using the program *psocake*^19,20^. First, one designs a mask for each run defining bad regions, usually blocking the zeroth order diffraction fringe, pixels too far away from the diffraction center and other “bad” regions in the detector where there is significant instrument scattering or there are readout issues with specific pixels. The total photon numbers in the remaining region is calculated, and then patterns are sorted according to the total photon counts per frame as shown in Figs. 6, 7, 8, and 9. The threshold at which to stop accepting frames is then determined by inspection of individual data frames from high intensity to lower intensity. Below a certain number of photons in the region of interest, the diffraction fringes are no longer visible. When diffraction fringes are no longer visible by eye, the image is considered to contain not enough data to be classified as a hit and is classified as empty or blank for preliminary processing. Frames with higher total photon counts than that value are considered hits and retained for subsequent analysis.

Not all the patterns retained above are valid diffraction patterns from a single PR772 virus particle. These patterns are further classified manually to select the single-hit patterns, from those consisting of clusters of PR772 virus particle. This clustering occurs when two or more PR772 virus particles are contained in an single aerosolization droplet causing the viruses to stick together. A trade off between higher isolated particle hit rates and a higher number of clusters is observed as increasing hit rates to higher levels usually requires changing sample concentration or GDVN conditions in the same direction that also increases the probability of multiple particles existing in an aerosolization droplet. It is acknowledged that this analysis process is influenced by human bias, however it is relatively straightforward to distinguish good single hit patterns from the others for PR772 particles when the intensity is high enough, because the PR772’s shell possess pseudo-icosahedral symmetry this lends itself to a distinct diffraction pattern at low diffraction angles.

## Data Records

**Fig. 10:**
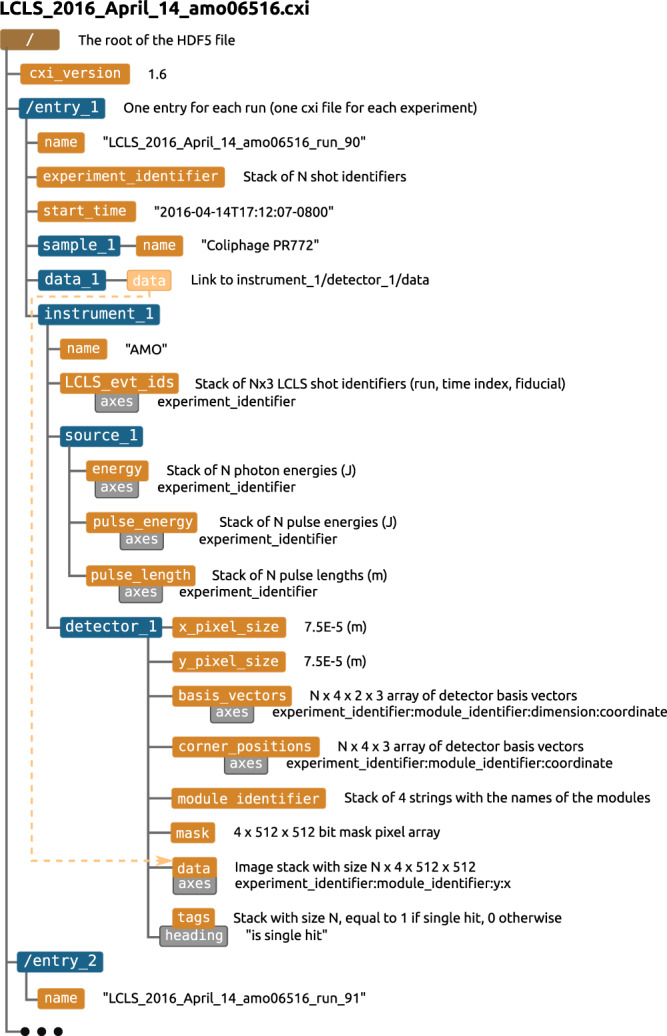
The structure of the CXI file containing the photon converted and selected diffraction data.

We provide access to the experiment data, both in the native file format used by the LCLS and in the CXI file format^21^. The LCLS stores beamtime data in the XTC format, which is optimised for sequential reading and writing. The XTC files contain the unprocessed “raw” detector data and metadata for every event in the selected experiment runs. Instructions for extracting data from XTC formatted files can be found at the LCLS data analysis website: https://confluence.slac.stanford.edu/display/PSDM/LCLS+Data+Analysis. The CXI format is based on the popular HDF5 format, which is a self-describing container for multidimensional data structures. The CXI format can be understood as simply a set of conventions for storing scientific data relating to coherent x-ray imaging in a HDF5 file. The CXI files contain the processed and selected diffraction patterns following version 1.6 of the standard, as shown in Fig. 10. There is one cxi file per experiment. The data corresponding to the nth experiment run is stored in a separate “entry” /entry_n, for example, the data for run 90 of the AMO06516 experiment is stored in /entry_1 of the file amo06516.cxi, since this is the first run that has been selected from that experiment.

The pnCCD detector^16^ used to collect these data is composed of 2 panels, as stated above, with two readout electronic back-ends per panel (each containing 4 analogue to digital converters). Each readout is composed of a 2D pixel array of shape 512 × 512. In the stack format, the recorded image data, are presented in an array with a shape of (4, 512, 512). In this 3D array, the first index is the index of the electronic readout, and the last two are the indexes of a specific pixel in that panel. When one would like to represent the actual spatial arrangement of the pixels with a 2D array, one can use psana functions to assemble arrays in the stack format and obtain the corresponding array in the 2D format. Alternatively, one can use the the corner_positions and basis_vectors datasets to determine the x and y coordinates of each pixel, as documented in the CXIDB file description. In the CXI file, this diffraction data (after conversion to photon counts) is stored in the data set /entry_n/data_1/data, which is an N × 4 × 512 × 512 unsigned 16 bit integer dataset, where N is the number of frames in the experiment run.

In addition to the diffraction data, the datasets energy, pulse_energy and pulse_length contain the X-ray pulse properties, basis_vectors and corner_positions the detector geometry, mask the detector mask and tags the image classification labels (1 if the diffraction was deemed to have originated from an isolated PR772 molecule and 0 otherwise). For a detailed explanation of these datasets, see the version 1.6 format description at^21^.

### Data access

All datasets described above are deposited in the Coherent X-ray Imaging Data Bank (CXIDB)^21^ in the CXIDB data format^7^.

### Data statistics

The run number range, total hit number, single hit number and the single hit to total hit number ratio are summarized in Table 1 The hit threshold, the number of measured photons required to be classified as a “hit”, for amox34117 has been set to a lower value, compared to the other experimental runs, which causes the drastic drop in the single to total hit number ratio.

The detailed distribution of total hits and single hits during each run are summarized in Table 2.

**Table 2:** Summary of experiment conditions and dataset statistics.

AMO87215													
Run	49	54	55	56	57	58	59	60	61	62	63	64	65
Single	0	0	8	4	5	7	71	14	139	126	320	378	324
Total	5	1	36	25	112	37	591	239	1182	700	1439	1678	1186
Run	66	67	68	69	71	72	73	74	75	76	77	78	
Single	160	33	96	5	6	3	1	171	163	172	58	203	
Total	487	115	935	208	564	206	78	365	265	326	93	573	
AMO06516													
Run	90	91	93	94	95	96	97	99	100	101	102	104	105
Single	106	101	12	60	22	475	128	70	189	200	29	67	300
Total	1122	984	217	902	379	6850	1938	1009	1396	2723	289	900	3238
Run	106	107	108	109	111	113	114	116	117	118	119	121	122
Single	74	481	484	409	461	3	376	487	438	406	375	432	410
Total	708	4681	4711	4155	4088	26	3028	3759	3592	3404	3022	2945	3364
Run	123	124	126	127	128	129	132	133	137	138	143		
Single	355	385	355	350	369	13	395	201	0	6	9		
Total	3373	2705	2511	4009	3786	287	2716	1681	1	26	71		
AMO11416													
Run	38	42	44	45	46	47	48	49	50	55	56		
Single	1	1	1	0	0	0	6	83	119	128	135		
Total	964	257	368	117	121	3	190	1232	1294	2336	1324		
AMOX34117													
Run	130	131	132	133	134	135	136	141	147	148	149	150	151
Single	18	19	19	2	4	25	1	0	0	0	0	1	0
Total	379	507	521	108	280	1598	126	111	460	494	165	1570	1044
Run	152	153	154	155	156	157	158	159	160	163	164	165	168
Single	0	0	0	0	0	0	0	0	0	0	0	0	0
Total	194	351	376	750	1437	61	231	94	114	1.6e4	2.5e4	2.5e4	1052
Run	169	170	172	173	174	175	176	177	178	179	180	181	182
Single	0	0	0	0	0	0	10	6	3	3	0	1	16
Total	343	104	131	86	223	698	4749	3131	1032	2338	1196	1850	6191
Run	183	184	185	186	187	188	189	190	191	192	193	194	200
Single	70	5	61	65	119	3	4	13	4	6	1	2	0
Total	3532	980	2070	976	7466	1.5e4	1.0e4	8007	4350	4252	2601	1523	643
Run	201	202	203	204	205	206	209	210	211	212	213	214	215
Single	0	0	0	0	0	2	2	5	17	15	0	0	0
Total	1209	1484	714	6300	5841	26	354	79	402	322	19	423	989
Run	216	217	218	219	220	221	222	225	226	227	228	229	230
Single	0	0	0	0	0	0	0	7	86	59	79	96	164
Total	273	266	152	374	128	134	10	315	1078	1170	1043	2831	2909
Run	231	232	233	234	235	236							
Single	63	33	42	17	139	86							
Total	1267	284	574	396	2388	2141							

## Technical Validation

**Fig. 11:**
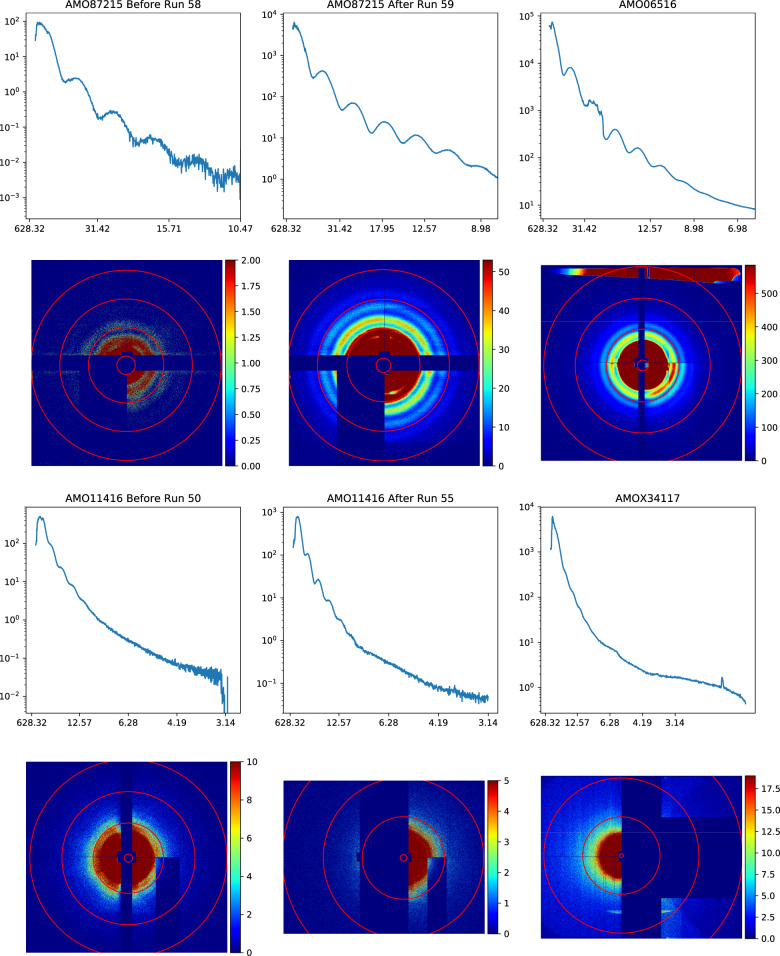
Pseudo SAXS patterns for six different configurations; (first and third rows) pseudo 1D SAXS profile, with the x-axis scaled to resolution in nm, and the y-axis in arbitrary units. (second and fourth rows) 2D summed SAXS patterns from single-hits after mapping the detector panels to x-y coordinates in the laboratory frame. Note: the red circles are to show the center of the pattern and the tile locations and not resolution. As all of the images are of the same size PR772 virus capsid the resolution of the diffraction speckle fringes is an indication of the camera length and hence resolution.

As a measure of the reliability of the datasets, all single-hits from each experiment were summed to form pseudo small angle X-ray scattering (SAXS) patterns (see the first and third rows of Fig. 11). These SAXS patterns are calculated as a function of resolution, accounting for the missing diffraction data and changing detector distance in each dataset, thus one can compare the SAXS profiles across the 6 groups of data.

The second and fourth rows of Fig. 11 show the 2D summed images corresponding to each of the 1D pseudo SAXS profiles. In these summed patterns background and detector artifacts are observable. It is noted that for amo87215 one of the panels had an issue with the readout electronics so that two of the analogue to digital converters read out at a different gain levels. For amo06516 there was a gap in the scatter shield of the second aperture, resulting in an increased level of beamline background signal in the unshielded area, located on the side of the detector (upper part of the image). For amo11416 an analogue to digital converter readout gain issue, similar to amo087215, is also observed. Additionally after run 55 one can observe the increase in the gap of the detector to allow one of the panels to obtain higher resolution. For amox34117 the center four of the analogue to digital converters readouts on one of the panels were not operational.

## Usage Notes

The dataset contains the recorded data during the experiment in both XTC and CXIDB formats. The dataset also contains a set of pre-selected hits and metadata as described in this paper. XTC files are the native format of LCLS and can be read using analysis frameworks provided by the LCLS (see https://confluence.slac.stanford.edu/display/PSDM/LCLS+Data+Analysis.

## Data Availability

Instructions for downloading and installing *psana* can be found: https://confluence.slac.stanford.edu/display/PSDM/Offsite+Installation.

## References

[1] Aquila A (2015). The linac coherent light source single particle imaging road map. Structural Dynamics.

[2] Seibert MM (2011). Single mimivirus particles intercepted and imaged with an x-ray laser. Nature.

[3] Ekeberg T (2016). Single-shot diffraction data from the mimivirus particle using an x-ray free-electron laser. Scientific Data.

[4] Benner WH (2008). Non-destructive characterization and alignment of aerodynamically focused particle beams using single particle charge detection. Journal of Aerosol Science.

[5] Hantke MF (2014). High-throughput imaging of heterogeneous cell organelles with an x-ray laser. Nature Photonics.

[6] Reddy HK (2017). Coherent soft x-ray diffraction imaging of coliphage pr772 at the linac coherent light source. Scientific data.

[7] Morgan AJ (2020). Coherent X-ray Imaging Data Bank.

[8] Neutze R, Wouts R, van der Spoel D, Weckert E, Hajdu J (2000). Potential for biomolecular imaging with femtosecond x-ray pulses. Nature.

[9] DePonte DP (2008). Gas dynamic virtual nozzle for generation of microscopic droplet streams. Journal of Physics D: Applied Physics.

[10] Weierstall U, Spence JCH, Doak RB (2012). Injector for scattering measurements on fully solvated biospecies. Review of Scientific Instruments.

[11] Nazari R (2020). 3d printing of gas-dynamic virtual nozzles and optical characterization of high-speed microjets. Optics Express.

[12] Ferguson KR (2015). The atomic, molecular and optical science instrument at the linac coherent light source. Journal of Synchrotron Radiation.

[13] Osipov T (2018). The lamp instrument at the linac coherent light source free-electron laser. Review of Scientific Instruments.

[14] Bozek JD (2009). Amo instrumentation for the lcls x-ray fel. The European Physical Journal Special Topics.

[15] Moeller, S. et al. Photon beamlines and diagnostics at lcls. Nuclear Instruments and Methods in Physics Research Section A: Accelerators, Spectrometers, Detectors and Associated Equipment **635**, S6–S11, 10.1016/j.nima.2010.10.125 PhotonDiag 2010. (2011).

[16] Strüder L (2010). Large-format, high-speed, x-ray pnccds combined with electron and ion imaging spectrometers in a multipurpose chamber for experiments at 4th generation light sources. Nuclear Instruments and Methods in Physics Research Section A: Accelerators, Spectrometers, Detectors and Associated Equipment.

[17] Meidinger, N. *et al*. pnccd for photon detection from near-infrared to x-rays. Nuclear Instruments and Methods in Physics Research Section A: Accelerators, Spectrometers, Detectors and Associated Equipment **565**, 251–257, 10.1016/j.nima.2006.05.006, Proceedings of the International Workshop on Semiconductor Pixel Detectors for Particles and Imaging (2006).

[18] Philipp HT, Koerner LJ, Hromalik MS, Tate MW, Gruner SM (2010). Femtosecond radiation experiment detector for x-ray free-electron laser (xfel) coherent x-ray imaging. IEEE Transactions on Nuclear Science.

[19] Damiani D (2016). Linac coherent light source data analysis using psana. Journal of Applied Crystallography.

[20] Thayer J (2017). Data systems for the linac coherent light source. Advanced structural and chemical imaging.

[21] Maia FRNC (2012). The coherent x-ray imaging data bank. Nature Methods.

